# Pesticide residue exposure provides different responses of the microbiomes of distinct cultures of the stored product pest mite *Acarus siro*

**DOI:** 10.1186/s12866-022-02661-4

**Published:** 2022-10-19

**Authors:** Jan Hubert, Blanka Navratilova, Bruno Sopko, Marta Nesvorna, Thomas W. Phillips

**Affiliations:** 1 Crop Research Institute, Drnovska 507/73, 16106 Prague 6 – Ruzyne, Czechia; 2 Department of Microbiology, Nutrition and Dietetics, Faculty of Agrobiology, Food and Natural Resources, Czech University of Life Sciences Prague, Kamycka 129, 165 00 Prague 6 – Suchdol, Czechia; 3 Department of Ecology, Faculty of Science, Charles University, Vinicna 1594/7, CZ-128 44 Prague 2 – New Town, Czechia; 4 Department of Entomology, Kansas State University, Manhattan, KS 66506 USA

**Keywords:** Pesticide; Storage; Interaction; Tolerance; Symbionts

## Abstract

**Background:**

The contribution of the microbiome to pesticide breakdown in agricultural pests remains unclear. We analyzed the effect of pirimiphos-methyl (PM) on four geographically different cultures of the stored product pest mite *Acarus siro* (6 L, 6Tu, 6Tk and 6Z) under laboratory experiments. The effect of PM on mite mortality in the impregnated filter paper test was compared.

**Results:**

The mite sensitivity to PM decreased in the order of 6 L, 6Tu, 6Tk, and 6Z. Then, the mites were cultured on PM residues (0.0125 and 1.25 µg·g^−1^), and population growth was compared to the control after 21 days of exposure. The comparison showed two situations: (i) increasing population growth for the most sensitive cultures (6 L and 6Tu), and (ii) no effect on mite population growth for tolerant cultures (6Z and 6Tk). The microbiome of mites was analyzed by quantification of 16S DNA copies based on quantitative polymerase chain reaction (qPCR) and by barcode sequencing of the V4 fragment of 16S DNA on samples of 30 individuals from the control and PM residues. The microbiome comprised primarily *Solitalea*-like organisms in all cultures, except for 6Z, followed by *Bacillu*s, *Staphylococcus*, and *Lactobacillus.* The microbiomes of mite cultures did not change with increasing population density. The microbiome of cultures without any differences in population density showed differences in the microbiome composition. A *Sodalis*-like symbiont replaced *Solitalea* in the 1.25 µg·g^−1^ PM in the 6Tk culture. *Sodalis* and *Bacillus* prevailed in the microbiomes of PM-treated mites of 6Z culture, while *Solitalea* was almost absent.

**Conclusion:**

The results showed that the microbiome of *A. siro* differs in composition and in response to PM residues in the diet. The results indicate that *Sodalis*-like symbionts can help recover mites from pesticide-induced stress.

**Supplementary Information:**

The online version contains supplementary material available at 10.1186/s12866-022-02661-4.

## Background

Pests in durable stored agricultural products have been recognized as a serious threat to food supplies for many centuries. Grain stored at farms and value-added grain products in food processing plants share characteristics that put them at risk of pest infestation, including high relative humidity resulting in storage of damp grain, poor air circulation, scarification of the grain during harvest and storage manipulations, water seepage, and accumulation of plant debris, which are all factors contributing to massive mite and insect infestations [1]. Even though stored grain mites (Sarcoptiformes: Acaridae) do not attack the grain when the coat is intact [2], during threshing and storage manipulations, more than 90% of the grain is scarified and therefore can be destroyed by the mites. Grain mites contaminate stored products introduced from fields with their feces, with allergens, and by the transfer of mycotoxin-producing fungi [3]. *Acarus siro* L. is the dominant species in stored product habitats of cosmopolitan occurrence [4], feeding directly on grains [4, 5], various grain products, dried cheeses [6], and various fungi [6, 7].

The most common method for controlling stored product mites is the use of pesticides [8–10]. However, none of the pesticides is labeled against stored mites but for stored product insects. However, the pesticide is expected to control stored product mites [8, 11]. In the last 80 years, massive pesticide applications in cheese-producing factories have led to the development of resistance to the organophosphate insecticides etrimfos [12] and pirimiphos-methyl [13] and the chlorinated hydrocarbon lindane [14] in *A. siro*. Resistance to pesticides in *A. siro* likely occurred via metabolic detoxification caused by high esterase activity, but it is probable that other mechanisms were also involved [13]. However, previous studies did not determine the origin of such enzymes regarding whether they are endogenous, produced by mites, or possibly originate from their associated gut microflora [15, 16]. Due to the transfer of mites with the commodities, the spreading of resistance was expected, but to date, the resistance of stored product mites has not been studied.

Microbiome-facilitated resistance to pesticides was reported more than 50 years ago, when an obligate extracellular bacterial symbiont, *Pseudomonas melophthora*, of the apple maggot, *Rhagoletis pomonella* (Walsh), exhibited the capability to degrade chlorinated hydrocarbons, organophosphates, and carbamates through powerful esterase activity [17]. Various approaches to describing microbiome-induced resistance have recently been developed, ranging from molecular investigation to testing the influence of host diet on microbial communities [18]. Often, multiple bacterial species can degrade the same pesticide, which widens the spectrum of organisms that may be involved [19]. Moreover, endosymbionts may interfere with virus infections spread by insects [20], since some symbionts, such as *Wolbachia,* have shown antiviral effects in protecting their host from diverse viruses [21]. Intracellular symbionts commonly increase host susceptibility to chemical insecticides; however, there are reports of increased resistance [22].

Closer examination of microbial composition in *A. siro* identified *Bartonella*-like bacteria, intracellular symbiont *Cardinium* bacteria, *Solitalea*-like symbionts based on cloning and Sanger sequencing and comparison to identified sequences in GenBank [23–25]. Based on barcode sequencing, the microbiome of *A. siro* was found to include *Bartonella*-like, *Solitalea*-like, *Bacillus* sp., and *Kocuria* sp. [26, 27]. Previous studies on the related species *Rhizoglyphus robini* showed that their microbiomes differ among the different cultures of the same species [28]. Recently, we observed that pesticides influence the microbial profiles of different stored product mite *Tyrophagus putrescentiae* cultures; however, the effect of pesticides is not systemic [29].

In this study, we analyzed four different cultures of *A. siro*. We compared the microbiome profiles of mite cultures under controlled conditions. Then, we compared the sensitivity of mite cultures to pirimiphos-methyl (PM) in impregnated filter paper. In the next step, the mites from different cultures were placed on residual (under the recommended dose) PM concentrations in the diet. The microbiome was compared among the mite cultures and PM treatments after 21 days of mite exposure. The quantification of 16S DNA copies based on quantitative polymerase chain reaction (qPCR) and by barcode sequencing of the V4 fragment of 16S DNA were used to describe the microbiome changes.

## Results

### The difference in microbiome composition among A. siro cultures under controlled conditions

**Fig. 1:**
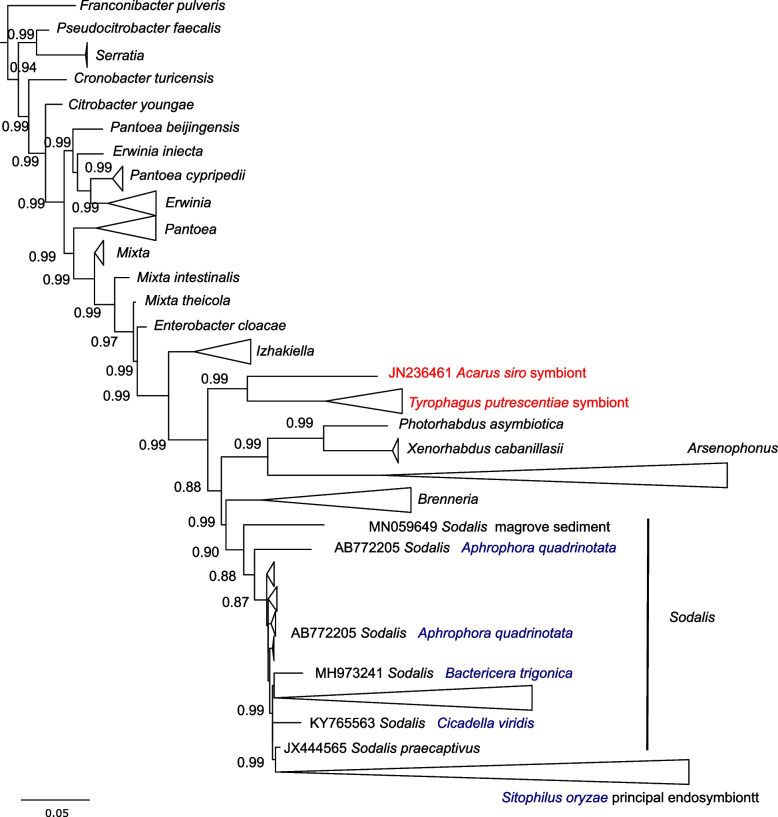
The identification of *Sodalis*-like symbionts of *Acarus siro* based on comparison of almost complete 16S DNA sequences from GenBank and clones from *A. siro* and *Tyrophagus putrescentiae* [25]. The figure shows a subtree including mite and insect symbionts and related clusters. Branch lengths correspond to mean posterior estimates of evolutionary distances (scale bar 0.05). Branch labels indicate supporting bootstrap values. The tree was rooted in *Escherichia coli* (U000096). The sequences and their descriptions are provided in Table S3, Supporting Information

The microbiome of *A. siro* was composed of 437 OTUs (operational taxonomic units at a 97% similarity level) (Table S2). However, a few OTUs were abundant in mite microbiomes, i.e., the first 10 most abundant OTUs contained 90% of the total read numbers (Table S3). Two symbionts (*Solitalea*-like OTU1 and *Sodalis*-like OTU3) were identified in the mite microbiome. Identification of the *Solitalea*-like symbiont was based on previous analyses of almost complete 16S DNA clones that clustered as a sister group of *Solitalea* bacteria outside members of the Sphingobacteriacea [30]. This Enterobacteriaceae symbiont had been identified previously in *A. siro* based on Sanger sequencing of cloned 16S DNA [25]. The 16S DNA sequences (JN236461) formed from the symbiont clones of the stored product mite *T. putrescentiae* cluster separately with *Photorhabdus* and *Xenorhabdus.* Thus, this cluster is outside the *Arsenophonus, Breneria*, and *Sodalis* sequences (Fig. 1).

**Fig. 2:**
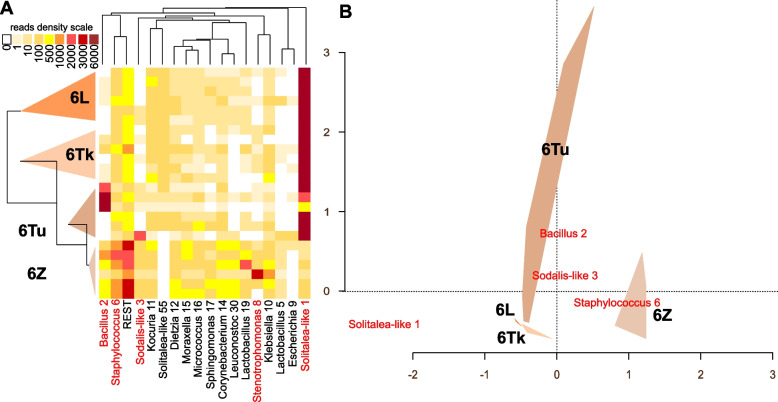
The comparison of differences in the microbiomes of *Acarus siro* cultures that were not treated with pirimiphos-methyl (control). **A** shows the heatmap of standardized relative abundances of bacteria in mites from the four cultures. The lengths of triangles on the left side of the heatmap indicate the variability in the samples from the same culture. A similar level of variability and microbiome similarity is seen in the OTU clusters at the top (uncondensed intro triangles). Both clusters (samples and OTUs) were constructed using the unweighted pair group method with arithmetic mean (UPGMA) in Bray–Curtis distance. The red color in OTUs indicates high variability explained in SIMPER analyses. **B** is a triplot of a distance-based redundancy analysis (dbRDA) indicating the position of samples according to ax1 and ax 2. The samples from the same culture are organized as convex hulls, and the most important bacterial OTUs are shown. Ax1 and ax2 explained 53% and 8% of the variability in the model, respectively

The mite populations differed in the composition of their microbiome profiles (dbRDA: R^2^ = 0.656, population: F(_3,18)_ = 10.425, *p* = 0.001, mite density: F_(1,18)_ = 1.296, *p* = 0.268). The 6 L and 6Tk populations were similar but differed from the 6Z and 6Tu populations (Fig. 2). The microbiome of population 6Tu showed high variability caused by different read numbers of *Solitalea*-like symbionts (OTU1). The 6 L and 6Tk samples were characterized by a high proportion of *Solitalea* (OTU1). *Solitalea*-like (OTU1) reads accounted for more than 90% of the 6L and 6Tk population bacterial profiles.

Culture 6Tu was characterized by *Solitalea*-like bacteria (OTU1), which formed 60% of the total reads in the microbiome profile, followed by *Bacillus* (OTU2) (30% of reads) and *Sodalis*-like symbionts (OTU3) (approximately 5% of reads). Culture 6Z showed a high diversity of OTUs, with the most abundant being *Staphylococcus* (OTU6), *Stenotrophomonas* (OTU8), an unidentified Enterobacteriaceae (OTU10), and *Lactobacillus* (OTU19); *Solitalea*-like (OTU1), *Sodalis*-like (OTU3) and *Bacillus* (OTU2) profiles were present in the microbiome, although they showed a very low level of reads compared to the other numbers (Fig. S1).

**Fig. 3:**
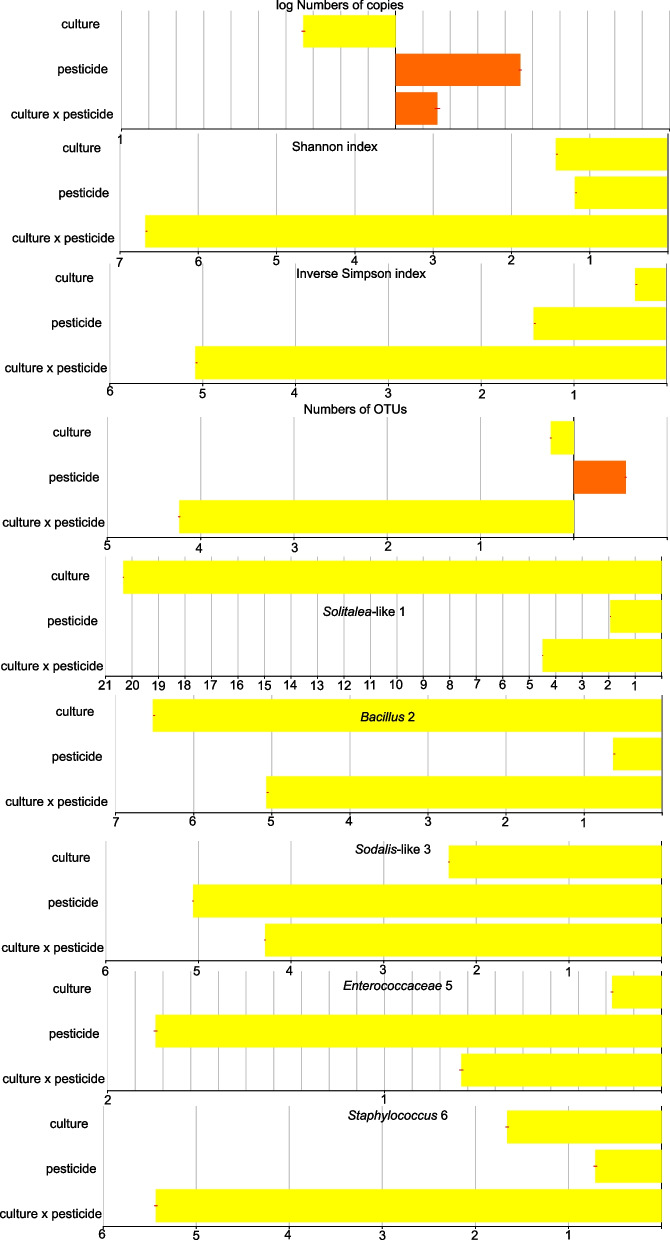
The Bayesian analyses of the contribution of pesticide and mite culture to *Acarus siro* microbiome composition. Using Bayesian statistics, we executed the full Bayesian ANOVA model and then ran the same model, omitting the factors one by one. The size of the bars at the x-axis (logarithmic scale) indicates how much the model fits worse when the corresponding predictor is dropped. The positive interactions are indicated by yellow, while negative interactions are indicated by brown

The Bayes analyses (Fig. 3) revealed that culture had some effect on the numbers of bacterial copies in comparison to the effect of pesticides or pesticide x culture interaction. The numbers of bacterial copies ranged between 10^3^ and 10^4^ per mite based on qPCR in all control samples. In contrast, both analyzed diversity indices (Table S5) were marginally influenced by mite culture (Fig. 3). The mite culture was the prevailing factor on PM and culture x PM interactions to explain variability in *Solitalea*-like (OTU1) and *Bacillus* (OTU2).

### Pirimiphos-methyl toxicity

**Fig. 4:**
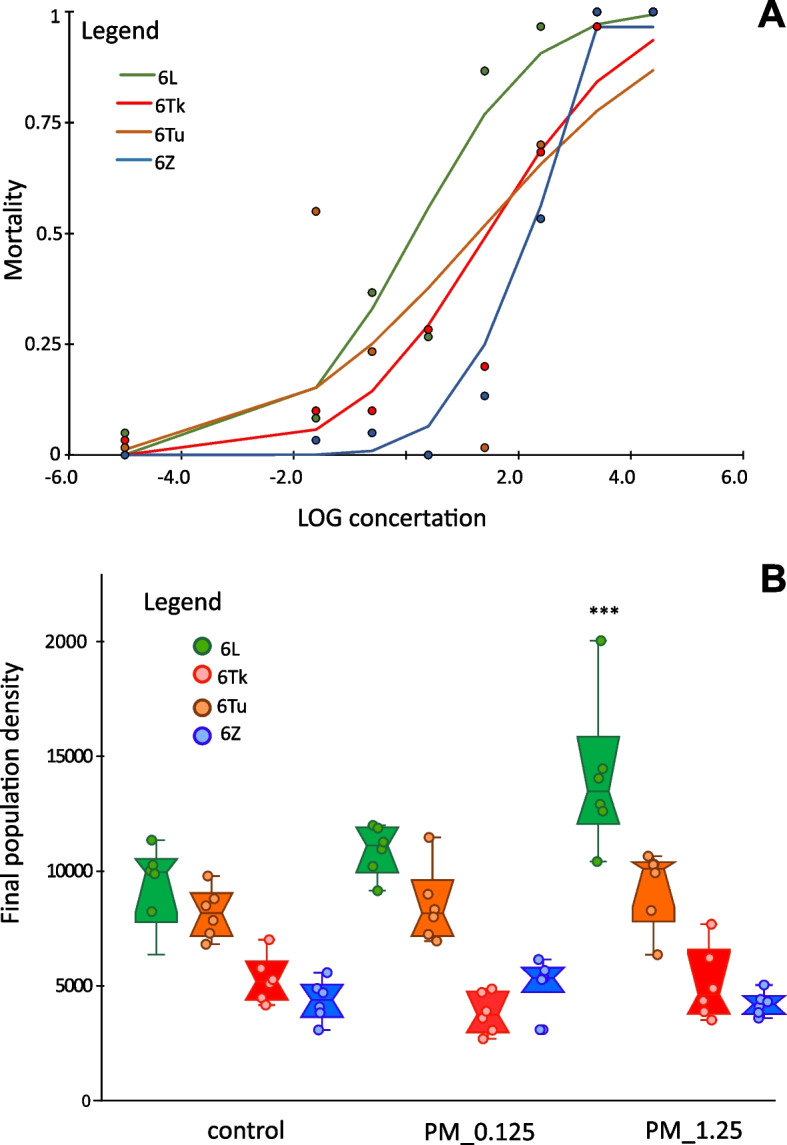
The effect of pirimiphos-methyl on cultures of *Acarus siro*. **A**—Mortality of mites in the impregnated filter paper test. The lines are model fits (for parameters, see Table 1) and points of observed values; **B**—The comparison of mite final population density after 21 days of growth on control and pirimiphos-methyl residues (1.25 µg and 0.0125 µg). The data are shown as jitter box plots. The significant differences (Tukey`s pos hoc comparison for interaction) among the control and PM for same culture are indicated by Asterix

PM was toxic to *A. siro* mites in the impregnated filter paper test; however, mortality differed among the mite cultures (GLM: culture model Chi = 433, *p* < 0.001, concentration = 547.34, *p* < 0.001 and interaction Chi = 377). The sensitivity to PM increased from 6 L, 6Tu, 6Tk and 6Z (Table 1; Fig. 4).

**Table 1:** The results of pirimiphos methyl aplication to mortality of *Acarus siro* cultures in impregnated filter paper test. The reggression parameters are provided and back estimate of LC_50_ and LC_95_ with 95% confidence inervals. The fitted doses are µg/mL of pesticide

Factor	Estimate	Std. error	Z value	P	LC50		LC95	
6L	-0.061	0.076	-0.804	0.421	1.31	(0.94–1.83)	1,795	(1,184–2,272)
6Tk	-0.759	0.079	-9.601	< 0.001	4.25	(3.70–4.89)	38,432	(25.000–59.000)
6Tu	-0.617	0.078	-7.933	< 0.001	3.24	(2.82–3.73)	20,590	(13,000–32,000)
6Z	-1.231	0.088	-13.974	< 0.001	10.46	(9.04–12.11)	305,375	(191,000–487,000)
concentration	0.524	0.021	24.482	< 0.001

Population growth on pesticide residues was influenced by both mite culture and PM (two-way analysis of variance (ANOVA): culture F_(2,60)_ = 5.148, *p* = 0.006; pesticide F_(3,60)_ = 85.42 *p* < 0.001 and interaction F_(6,60)_ = 4.264 *p* = 0.001). Cultures 6 L and 6Tu were very fast-growing and had a two-fold higher growth rate than the slower-growing 6Z and 6Tk cultures (Fig. 4). The density of mites on 6Tk, and 6Z cultures was not influenced by PM residues in the diets, while culture 6 L were influenced by PM residues. Growth of the 6 L culture was higher on a diet with a PM than on the control, this trend was observed for 6Tu, but was not significant in pos hoc test (Fig. 4).

### The effect of pirimiphos-methyl residues in the diet on changes in A. siro microbial profiles

PM residues influenced the microbiome of *A. siro* differently according to the mite culture and pesticide residue concentration. The total distance-based redundancy analysis (dbRDA) model explained 45% of the variability in the microbial profile using mite population and pesticide as environmental variables (Table 2). Based on permutation tests, the mite culture showed a higher influence on the dataset than pesticide, but both were significant. The partial models resulting when mite populations were assessed separately showed that PM-methyl influenced the bacterial profile in 6Tk (1.25 µg·g^−1^) and 6Z (both concentrations), while no effect was observed for the 6L and 6Tu populations (Table 2).

**Table 2:** The results of distance based redundance analyses of the bacterial profiles of *Acarus siro* microbiome. The constrained and unconstrained variations are showed. The analyses include total model for all tested cultures and partial models for every culture separately. In total model the popualtion and pesticide concetrations inlcuding control were used as the factors. In pratial mdoels two pesticide concentration were used as the factors. The effect of factors was evalauted by permutation test (*N* = 999). The significant interactions are marked as bold

	**dbRDA**		**Permutation test**		
**Model**	**constrained**	**Unconstrained**	**Factor**	**F**	**Pr**
Total	0.4518	0.5482	population	**15.87**	**0.001**
			pesticide	**6.7797**	**0.001**
6L	0.2365	0.7635	PM 1.25 µg	2.639	0.052
			PM 0.0125 µg	2.0074	0.097
6Tk	0.8156	0.1844	PM 1.25 µg	**65.921**	**0.001**
			PM 0.0125 µg	0.428	0.566
6Tu	0.2182	0.7818	PM 1.25 µg	1.1832	0.31
			PM 0.0125 µg	2.725	0.076
6Z	0.3907	0.6093	PM 1.25 µg	**2.9443**	**0.041**
			PM 0.0125 µg	**6.6723**	**0.002**

**Fig. 5:**
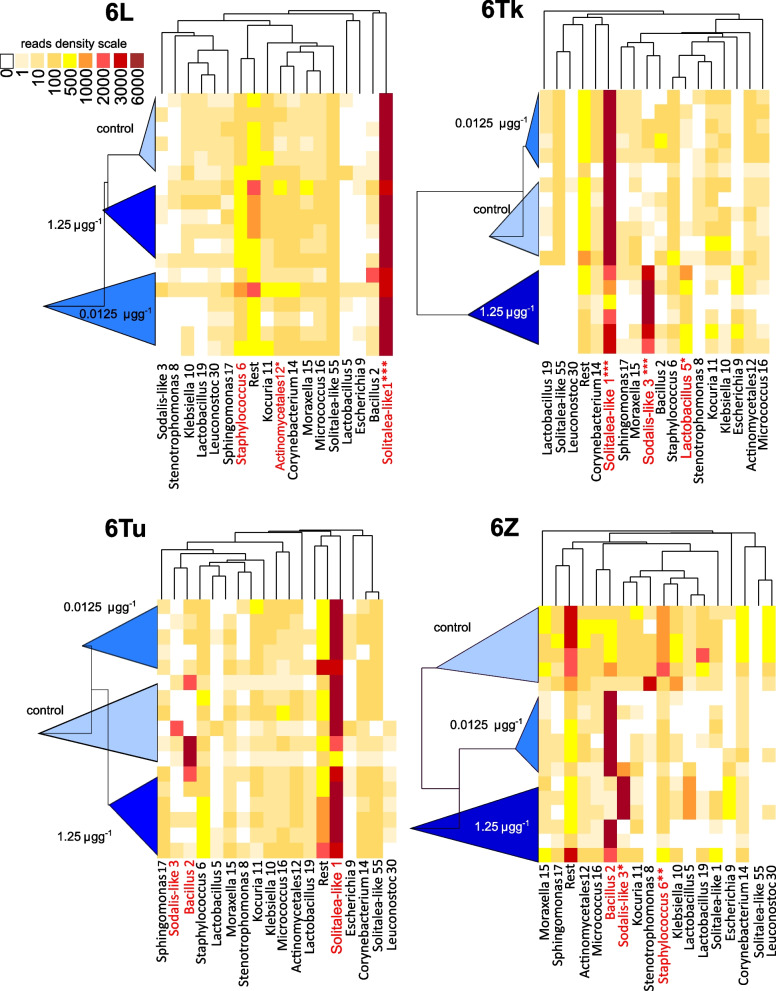
Comparison of the effect of pirimiphos-methyl residues on the microbiomes of *Acarus siro* cultures using heatmaps of standardized bacterial relative abundance. The lengths of triangles on the left side of the heatmap indicate the variability inside the samples from the same residues. A similar level of variability and microbiome similarity is seen in the OTU clusters at the top (uncondensed intro triangles). Both clusters (samples and OTUs) were constructed using the UPGMA method in Bray–Curtis distance. The red color in OTUs indicates high variability explained in SIMPER analyses, and the “*” indicates significant differences between control and 1.25 µg·g^−1^ pirimiphos-methyl concentration based on METASTATS (e.g., * *P* ≥ 0.01 and *P* < 0.05; ** *P* ≥ 0.001 and *P* < 0.01; *** *P* ≤ 0.001)

**Fig. 6:**
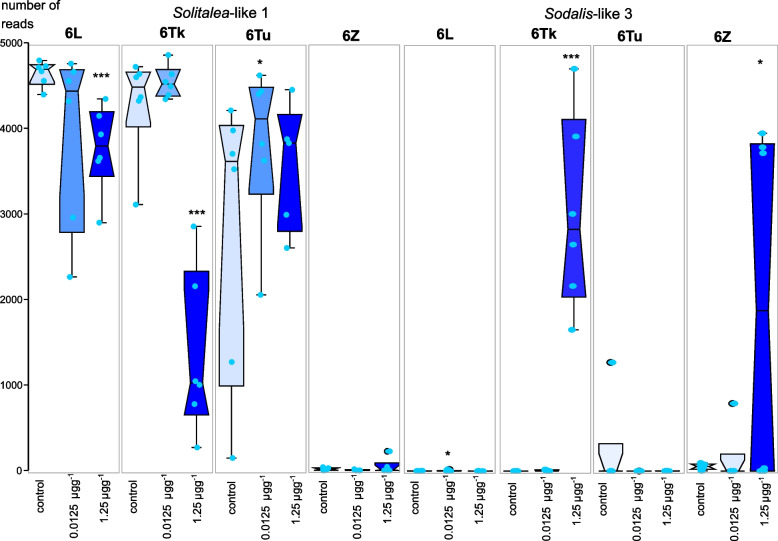
The numbers of reads of *Solitalea*-like and *Sodalis*-like bacteria in *Acarus siro* microbiomes from the untreated controls and cultures treated with three concentrations of pirimiphos-methyl in rearing diets. The graphs are jitter boxplots, and the asterisks indicate significant differences from the control based *on* METASTATS (e.g., * *P* ≥ 0.01 and *P* < 0.05; ** *P* ≥ 0.001 and *P* < 0.01; *** *P* ≤ 0.001)

The main changes in the microbiomes were due to the presence (6Tk) or absence (6Z) of *Solitalea*-like symbionts (OTU1) (Fig. S2). The 6Tk culture showed a replacement of the *Solitalea*-like symbiont (OTU1) by *Sodali*s-like symbiont (OTU3) at the 1.25 µg level of pirimiphos-methyl residues (Fig. 5). Due to the absence of the *Solitalea*-like symbiont (OTU1) in 6Z, an increase in the *Sodalis*-like symbiont profile was observed in 3 samples of the 6Z culture (Figs. 5 and 6). The Bayes analyses (Fig. 3) showed that the *Solitalea*-like symbiont (OTU1) had a different response to the tested factors (mite culture, PM and their interaction) than the *Sodalis*-like symbiont (OTU3). The profile of the *Sodalis*-like symbiont (OTU3) was more influenced by PM than mite culture, and the opposite situation was observed for the *Solitalea*-like symbiont (OTU1).

## Discussion

This study showed that the microbiome of *A. siro* differs in its composition among observed mite cultures. The mite cultures also differed in their response to PM; the sensitivity to PM decreased in the order of 6L, 6Tu, 6Tk, and 6Z. The recommended dose of PM provided 100% mortality of mites in the impregnated paper test. The mite growth on PM residues (0.0125 and 1.25 µg·g^−1^) under the recommended dose showed two responses in the comparison control: (i) increasing population growth showed the most sensitive populations 6L and 6Tu, and (ii) no effect on mite population growth was observed on tolerant populations 6Z and 6Tk. The microbiome analyses showed no effect of PM residues on cultures with increasing population growth, but those without any response showed changes in microbiome composition.

The increase in the growth of sensitive cultures of *A. siro* on residual concentration PM should be explained as a stress agent. Stimulation of an organism is documented, and the organism is therefore provided with increased sensitivity to respond to changes in its environment and with increased efficiency to develop new or better mechanisms to fit a suboptimum environment (e.g., hormoligiosis [31]). Such a reaction is documented for mites. Mite species *Panonychus ulmi* and *Neoseiulus californicus* were proven to lay more eggs when they were exposed to sublethal doses of cypermethrin, imidacloprid, deltamethrin and thiacloprid, while at the same time, their lifespan shortened [32]. Increased fecundity of the common spider mite pest *Tetranychus urticae* was observed after thiacloprid, acetamiprid, and thiamethoxam treatment [33]. Previously, we observed that 6L cultures of *A. siro* were tolerant to chlorpyrifos residues, and the population growth of these mites was greater in the chlorpyrifos-treated diets with concentrations of 10, 100 and 250 µg·g^−1^ compared to the control [34]. Our results indicated no connection between stress reactions and the microbiome in *A. siro* in these two cultures (6L and 6Tu).

No stress reaction caused by PM residues to two tolerant *A. siro* cultures (6Z and 6Tk) should be connected to microbiome changes. The results showed that the *Sodalis*-like (OTU3) symbiont replaced *Solitalea* (OTU1) in PM 1.25 µg·g^−1^ residues. *Sodalis* (OTU3) and *Bacillus* (OTU2) prevailed in the microbiome of PM residues for 6Z mites, while *Solitalea*-like bacteria were absent from that strain. Changes in the microbiome should be either stochastic or PM-induced. Because we did not observe any stochastic effect on cultures 6L and 6Tu regarding their microbiome profiles remaining unchanged after PM treatment, we suggest that the effect of PM on 6Tk and 6Z is caused by the treatments with pesticide residues in the mite diet.

The *Solitalea*-like symbiont was described based on a comparison of cloned Sanger sequences of 16S DNA from *A. siro* and *T. putrescentiae* [30]. The clones formed a distinct cluster separated from the neighboring clusters of the genus *Solitalea* and of uncultured bacteria (living in amoebae or in sweet water or soil habitats) [30]. The bacteria in that study were localized in the digestive and reproductive tract of *A. siro* using fluorescence in situ hybridization (FISH) and in eggs by PCR with specific primers [30] and supported symbiotic association. In the current study, we found that *Solitalea*-like OTUs formed 95% of reads in the microbial profiles of 3 cultures, which is supported by recent results from our laboratory [26].

*Sodalis* bacteria can establish facultative relationships with insect hosts by invading a variety of cells and show a relationship with the bioluminescent bacterial species *Photorhabdus* [35]. This earlier finding is similar to our classification of the *Sodalis*-like symbiont of cloned 16S DNA sequences from *A. siro* (Fig. S1) [25]. The next related symbiont is the *Sodalis* symbiont found in the sucking plant pests *Bactericera trigonica* (Hemiptera: Psylloidea) [36] and *Aphrophora quadrinotata* (Hemiptera: Aphrophoridae) [37] and the stored grain pest weevils *Sitophilus zeamais* and *Sitophilus oryzae* (Coleoptera: Curculionidae) [38, 39]. *Candidatus* Sodalis melophagi, an insect symbiont that inhabits the blood feeding louse fly (*Melophagus ovinus*) at approximately 50% of flies in a population [40], is another example of *Sodalis* endosymbiosis. An ultrastructural study revealed the presence of *Sodalis*-like endosymbionts in the cells of the bacterium *Sulcia* that were inside the guts of the green leafhopper *Cicadella viridis* (Hemiptera, Cicadellidae) [41]. Therefore, *Sodalis*-like endosymbionts are common in and perhaps crucial for the success of several plant-feeding insect species to improve their nutrition and/or degrade some harmful compounds.

The spittlebugs (Auchenorrhyncha: Cercopoidae) are inhabited by the Bacteroidetes symbiont *Sulcia muelleri* and the betaproteobacteria symbiont *Zinderia insecticola*, which are both restricted to their own bacteriocytes [42]. The ancestral *Zinderia* symbiont has been replaced with a novel symbiont related to *Sodalis glossinidus* (Enterobacteriaceae) [42], which appears to be a situation analogous to the *Solitalea*–*Sodalis* replacement that we found in the 6Tk *A. siro* culture induced by pyrimiphos methyl residues in the diet. Such replacement is expected to be beneficial to the host mite. The analyses of the genome of *Sodalis* symbionts showed the beneficial effects of these symbionts to their host by providing nutrients, vitamins, and possibly nitrogen fixation. For example, the genome of *Sodalis* symbionts of the spittlebug *Philaenus spumarius* (Insecta: Cercopoidea) included pathways for the production of enzyme cofactors (e.g., biotin, folate, flavin, vitamin B6, ubiquinone, glutathione, heme, thiamine), fatty acids, phospholipids, purine and pyrimidine nucleotides, terpenoids and components of the bacterial cell wall [37]. Similarly, the *Candidatus* Sodalis baculum symbiont of *Henestaris halophilus* (Hemiptera: Lygaeoidea) produces amino acids and cofactors for its host [43]. In addition, *Sodalis* is able to recycle 10–15% of host nitrogen [44]. The production of such compounds and nitrogen recycling should be beneficial for mite survival under stress conditions. Similar interactions are possible in the *Sodalis*-like symbiont and the *A. siro* host. Additional research will be necessary to characterize the genome of both symbionts and their impact on mite fitness.

The different tolerances of cultures of other species of mites to pesticides have been documented previously [45, 46]. Our data indicate that the microbiome of the *A. siro* response differs among the cultures and microbiome composition and is more complex.

## Materials and methods

### Mites

The following four cultures of *A. siro*, each started with mites from different geographic regions of Czechia, were used in the experiments: **6L** originated from grain stores in Bustehrad, Czechia, collected by Eva Zdarkova in 1996; **6Tk** originated from contaminated horse feed in Teplice, Czechia, collected by Marta Nesvorna (MN) in 2015; **6Tu** originated from contaminated rabbit feed in Tuchomerice, Czechia, collected by MN in 2016; and **6Z** originated from rape seed oil and debris in Zvoleneves, Czechia, collected by MN in 2011. The four mite cultures were kept isolated from each other in the laboratory to prevent cross contamination. The mites were reared in 100-mL filter cap cell culture flasks (Cell Culture Flask T25, Eppendorf cat. No. 0030710029, Hamburg, Germany) placed in desiccator boxes over a saturated solution of potassium chloride to maintain 85% relative humidity (RH) at 25 ± 1 °C in darkness. The cultures were renewed monthly, and new cultures contained 0.3 ± 0.1 g of diet and 500 mites. The 150 g of diet consisted of 15 g of instant dry baker’s yeasts (Mauripan, Mauri, Balikesir, Turkiye) and 135 g of wheat germ. Both ingredients were mixed using a kitchen blender, and the mixture was then dried for 30 min at 70 °C to eliminate microbes in the diet. For the experiments, the adult mites were obtained from under the plug and the inside surface of the chambers using a brush under a dissection microscope.

### Pesticide used in the experiment

Pirimiphos-methyl (PM) is the active ingredient in the commercial formulation Actellic 50 EC (Syngenta, Basel, Switzerland). The pesticide is not labeled against mites but against stored product insects. However, PM is expected to control stored product mites [11]. The recommended application is 8 mL (4 g of PM) of pesticide (Actellic 50 EC; 500 g/L pirimiphos-methyl) per 1 t of grain [47] and 1–4 mL per m^−2^ (e.g. 0.5–2 g of PM) [48]. The pesticide was diluted in water to the following concentrations: 25,000, 2500, 250, 25, 2.5, 0.25, and 0.025 µg mL^−1^.

### Impregnated filter paper test

The impregnated filter paper test [12, 13, 46, 49, 50] was modified as described previously [49]. The test was run in glass weighing bottles (cat. No. 264.228.01, Vitrum, Prague, Czechia). Two pieces of filter paper were cut into round shapes to fill the bottom of a bottle, which was 2.2 cm in diameter (3.8 cm^2^). Then, 50 µL of pesticide was applied on the paper, and the application included the recommended dose (e.g. 1.25*10^–3^—1,250 µg/mL). The recalculated recommended dose is 50 µg of PM per 1cm^2^, e.g. 190 µg PM/vial. As a negative control, 50 µL of distilled water was applied. The bottles were opened for 30 min, and 10 adult unsexed mites were placed inside the chamber. The flasks were then closed with a glass lid. After 24 h of exposure, the mites [46, 49] were counted and checked to determine whether they were alive or dead using a dissection microscope. Every mite that was not in motion was stimulated by a very small painting brush with soft bristles for a few seconds, and if they did not move, they were pronounced dead. The experimental design included 10 replicates per concentration and mite culture. The natural mortality was as follows: 6L 5%, 6Z 0%, 6Tu 2%, and 6Tk 3%.

### Population growth on pirimiphos-methyl residues in the diet

The protocol used previously was adopted to compare the effect of pesticides on mite population growth [34, 46]. The rearing diet containing pesticide residues was prepared by mixing 10 g of diet with 5 mL of diluted PM at each of the concentrations 0.0125 and 1.25 µg·g^−1^ belonging to the recalculated recommended concentration (4 µg·g^−1^). Both substances were mixed and lyophilized to ensure homogenous distribution of the active pesticide in the diet [51]. The control was the diet mixed with water instead of pesticide solution. The diet (0.01 g) was placed in a filter cap cell culture flask. Then, 50 unsexed mites were added to each flask. The flasks were stored at 25 ± 0.5 °C at 85% RH in darkness. After three weeks (21 days), the flasks were filled with 35 mL of modified Oudemans fluid [52] (87 mL of 70% ethanol and 5 mL glycerol; the last compound, glacial acetic acid (8 mL), was not used to avoid DNA extraction) and the contents pof the flask were transferred into a Falcon tube. The flask was mixed, and 1 mL of the mixture was transferred into a Petri dish in three replicates and counted under a dissection microscope. The mean number of mites was recalculated per chamber. The mites were transferred back. The falcons were stored in a freezer prior to DNA extraction. We evaluated six replicates of all treatments. The expected outcome was that mite cultures susceptible to a given pesticide concentration would have smaller populations at the end of the 21-day incubation compared to cultures from the water-control diet and diets with lower pesticide concentrations. The generation time of closely related *Acarus farris* at 25 ± 0.5 °C at 85% RH is 18 days [53], indicating that the test was approximately for one generation.

### DNA isolation

The DNA was isolated from the samples of mites originating from the control, and two PM 0.0125 and 1.25 µg/g^−1^ residues belonged to the recommended application dose. The mites were removed from the flask using a micropipette and applied to a sterile 100-µL cell strainer (Model 15–1100, Biologix Group Limited). The mites were surface-cleaned by washing them in 0.47% sodium chlorine solution (bleach). The bleach solution was replaced by 96% ethanol. Then, the mites were moved into sterile glass Petri dishes. Next, 30 adult mites (one sample) were moved from the Petri dish to a sterile 1.5-mL tube (Eppendorf Safe-Lock Tubes, cat. No. 0030120086, Eppendorf Quality TM). The excess alcohol was removed using a micropipette. To ensure that the mites were completely dry, the tubes were placed into a SpeedVac Concentrator (SPD111 V, Thermo Scientific, Waltham, MA, USA) for 5–6 min. Following drying, a NucleoSpin® Tissue XS (MACHEREY–NAGEL Inc., Allentown, PA, USA cat. No. 740901.250) kit was used for DNA extraction. First, 180 µl of T1 buffer was added to the tube, and mites were homogenized using sterile Bel-Art® Disposable Pestles (cat. No. BAF199230001, Merck, Kenilworth, NJ, USA). Then, 20 µl of proteinase K was added to the mixture, and the tubes were placed in a preheated orbital shaking incubator (NB-205, N-Biotek, Pyeongcheon-ro, South Korea) for 60 min (56 °C, 300 rpm). Then, according to the manufacturer’s instructions, the samples were diluted with 30 µL of double distilled water and stored in a deep freezer prior to analysis. The experiments included six replicates for each treatment.

### qPCR and barcode analyses of the mite microbiome

The new protocol for sampling 30 mites per sample was developed in comparison to previous studies [29, 54, 55] using 1,000 of mites. The DNA was amplified with primers targeting the V4 variable region of the microbial 16S rRNA gene CS1_515Fc and CS2_806Rc primers [56] and containing 5’ linkers Fluidigm AccessArray linker sequences [57] in qPCR assay at Step One™ real time PCR (Thermo Fisher Scientific, Waltham, MA, USA). The master mix included primers, PCR H_2_O, and TP SYBR 2 × Master Mix (Top Bio, Vestec, Czechia). The PCR conditions were as follows: initialization at 95 °C for 2 min, denaturation at 95 °C for 10 s, annealing at 55 °C for 30 s, and elongation at 72 °C for 30 s repeated 40 times. The standard originated from *Melissococcus plutonius* sequenced by universal 27F/1492R primers [58]. The PCR products were purified with a Wizard SV Gel and PCR Cleanup Kit (Promega). The PCR products from bacterial primers were cloned using pGEM-T Easy Vector (Promega, Madison, WI, USA) and sequenced by the Sanger dideoxy method (Macrogen, Seoul, South Korea). The competent bacterial cells with plasmids were inoculated in LB medium (Himedia, Mumbai, India) with ampicillin 0.1 g/L (Cat No. A01104.0005, Duchefa Biochemie, Haarlem, The Netherlands) for 16 h at 37 °C. The plasmid was then purified with a Wizard® Plus SV Minipreps DNA purification system (Cat. No. A1330, Promega) according to the manufacturer’s protocol. Plasmids were linearized by SacI restriction (Cat. No. R6061, Promega) and cleaned with a Wizard SV gel and PCR Clean-Up system (Cat No. A9285). The concentration of the cleaned product was measured on a P330 Implen NanoPhotometer (Munich, Germany) and adjusted to 10 ng of DNA for each reaction. The DNA standard was diluted by 1/10 [54, 59]. The resulting numbers of copies were calculated per individual mite. Before analyses, gene abundance data were log_10_-transformed.

The resulting amplicons were then prepared for sequencing with second-stage PCR. During this second stage, Illumina sequencing adapters and sample-specific barcodes were incorporated into amplicons by PCR amplification with a Fluidigm Access Array for Illumina primers as described previously [26]. The barcoding PCR step and amplicon sequencing were performed at the Genome Research Core, Research Resources Center, University of Illinois (Chicago, IL, USA) on a MiniSeq platform (Illumina, San Diego, CA, USA) employing paired-end 2 × 153 bp reads. Raw sequences are available at the GenBank SRA under project PRJNA774490 (samples SAMN22566697—SAMN22566767; Table S1; details in Supporting Information). Forward and reverse sequences were processed in combination with the software MOTHUR [60, 61] and UPARSE [62, 63] according to previously described protocols [26, 55]. The OTUs (operational taxonomic units at 97% similarity identity) were identified using the sintax command in UPARSE [64] using the RPD training dataset [65]. The OTUs were also compared to those in GenBank [66] using blastn (Table S2). The unstandardized reads showed a minimum of 1,378, a maximum of 34,890, a mean of 16,292, and a median of 15,825 reads per sample (Table S3). The data were standardized per 5,000 reads/sample (Table S3) to obtain data comparable to previous analyses [55]. The diversity indices (inverse Simpson and Shannon) were calculated in PAST [67] for the standardized dataset.

The identification of *Sodalis*-like symbionts was based on two steps. First, out3 showed 95% similarity to cloned Sanger sequences of uncultured bacteria (JN236461) from *A. siro* [25] and 97–99% similarity to cloned Sanger sequences of uncultured bacteria from the mite *T. putrescentiae*. In the next step, we aligned 92 selected sequences of almost complete 16S RNA, including Sanger sequences from *A. siro* and *T. putrescentiae* (Table S4), using T-coffee [68, 69]. The alignment was processed in PhyML [70] and finalized in FigTree [71].

### Statistical analyses

The analyses were conducted in R 4.1.0 [72]. The mortality of mites in the impregnated filter paper test was analyzed using generalized linear models [73] using probit analyses of mortality [74] and factors (mite culture and PM concentration). The effect of factors was compared by the Chi square test. The LOG + 0.00001 transformation was applied to the PM concentration data. The MASS package was used for back estimates of LC_50_ and LC_95_ [75]. The effect of mite culture and pesticide (control, PM 1.25 µg and PM 0.0125 µg) on final mite density was analyzed by two-way analysis of variance (ANOVA) in PAST [67]. The data showed normal distribution. The Tukey's post hoc comparison was calculated for interactions.

The microbial community structure profiles were analyzed using the vegan package in R [76]. The effect of pesticide, pesticide concentration, mite culture, and mite density on the composition of the microbiome in the culture was analyzed using distance-based redundancy analyses (dbRDA) in Bray–Curtis distance, and the effect of variables (pesticide, mite culture, mite density) on OTUs was compared by a Monte-Carlo permutation test (999 permutations). Because all tested variables showed significant effects except mite density, we constructed models for mite cultures separately. The pesticide was the factor. Differences in the relative abundance of OTUs among the tested pesticide concentrations were compared using METASTATS (10,000 permutations) based [77] on MOTHUR software [60]. We also adapted similarity percentage (SIMPER) analyses to evaluate the contribution of particular OTUs to the dissimilarity among the treatments using PAST [67].

The effects of tested factors (mite culture, pesticide concentration and their interaction) on diversity indices, abundance of selected OTUs and numbers of copies from qPCR were analyzed by a general Bayesian test (package “BayesFactor”) [78]. Using Bayesian statistics, we executed the full Bayesian ANOVA model and then ran the same model, omitting the factors one by one. The explained variability was compared on a logarithmic scale.

## Supplementary Information

**Additional file 1: Fig. S1.** Microbiome profile of the cultures of *Acarus siro* in the control conditions based on barcode sequencing.

**Additional file 2: Fig. S2.** Microbiome profiles of the cultures of *Acarus siro* in the control conditions and under pirimiphos-methyl residues based on barcode sequencing. The columns are means from 6 replicates.

**Additional file 3: Table S1.** The list of samples, factors and files deposited at NCBI (PRJNA774490) describing the microbiome of *Acarus siro*. **Table S2.** Identification of OTUs based on RDP and comparison to GenBank using Blatstn in *Acarus siro* microbiome. The identified taxonomy levels and thresholds (Tr) are shown. The most similar hits in GenBank are presented (Blast hits) with their percentual similarity to compared sequences (ident %). **Table S3.** The list of samples with standardized read numbers of *Acarus siro* microbiome, the total numbers of standardized reads (N) and frequency in the samples (F) are provided. **Table S4.** The list of almost complete 16S DNA sequences used for the identification of *Sodalis*-like symbiont of *Acarus siro*. **Table S5.** The list of samples of *Acarus siro* microbiome, diversity indexes and numbers of reads based on qPCR (universal primers). The reads were recalculated per mite.

## Data Availability

Raw sequence data were submitted to the Sequence Read Archive (SRA) at the National Center for Biotechnology Information (NCBI) website under the accession number PRJNA774490. The datasets generated during and/or analyzed during the current study are available as supplementary datasets.
