# Supplementary figures and images for: Pesticide residue exposure provides different responses of the microbiomes of distinct cultures of the stored product pest mite Acarus siro
Source: BMC Microbiol. 2022 Oct 19;22:252. doi: 10.1186/s12866-022-02661-4 (PMC9580201; doi:10.1186/s12866-022-02661-4)

## Cultures

## Reads profile

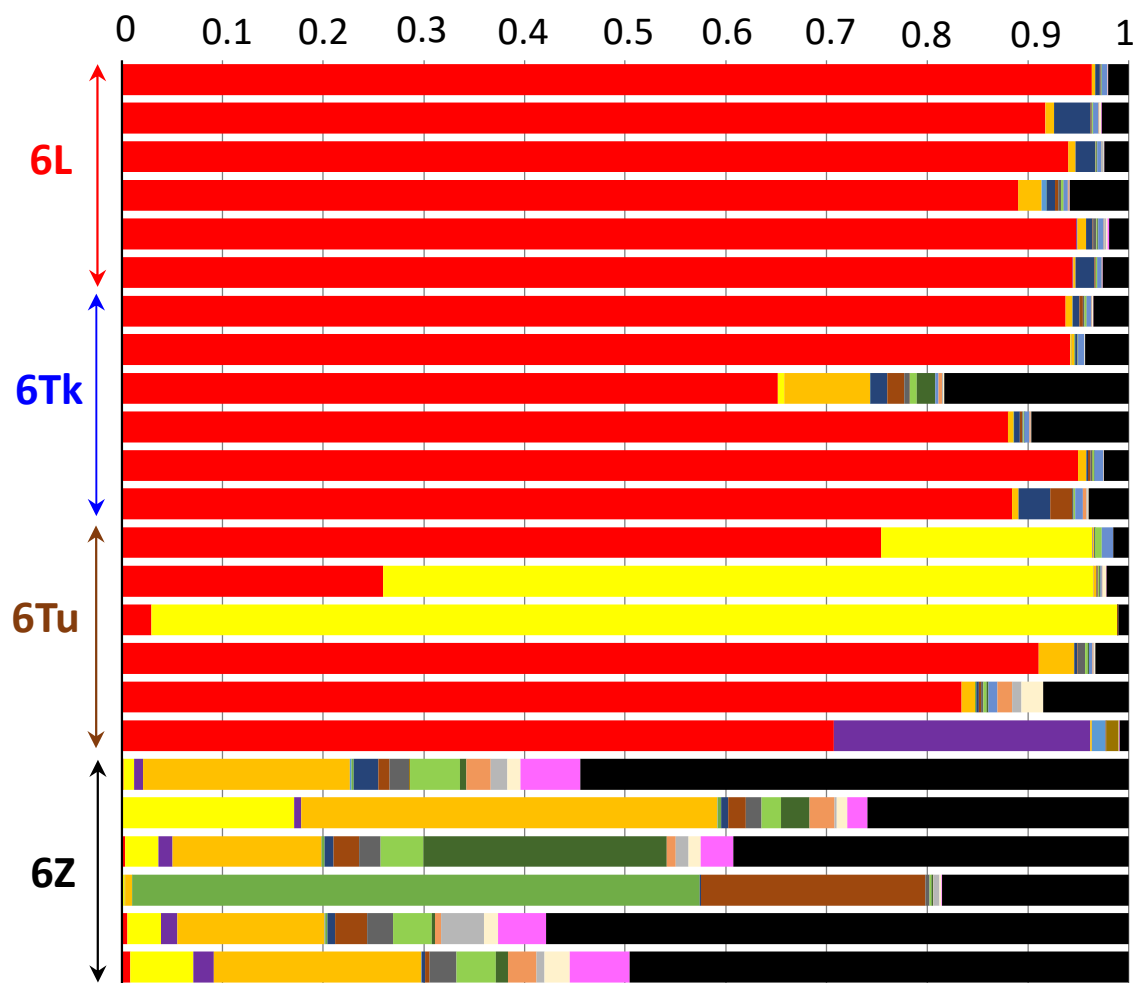

## Legends -OTUs

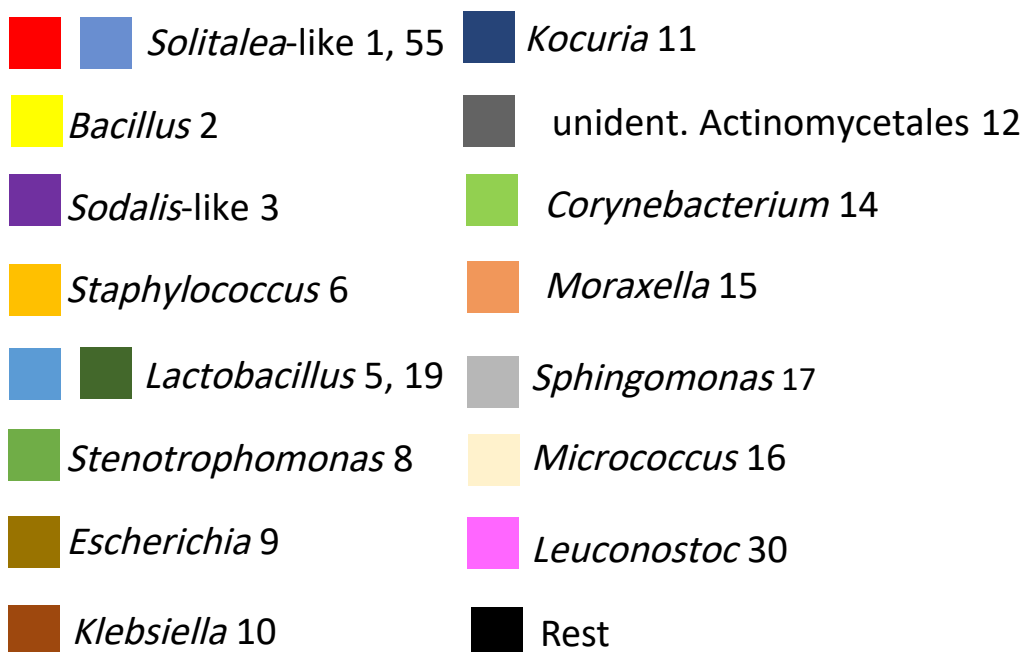

Supplement: Supplementary file 1 — Additional file 1: Fig. S1. Microbiome profile of the cultures of Acarus siro in the control conditions based on barcode sequencing. [file 12866_2022_2661_MOESM1_ESM.pdf]

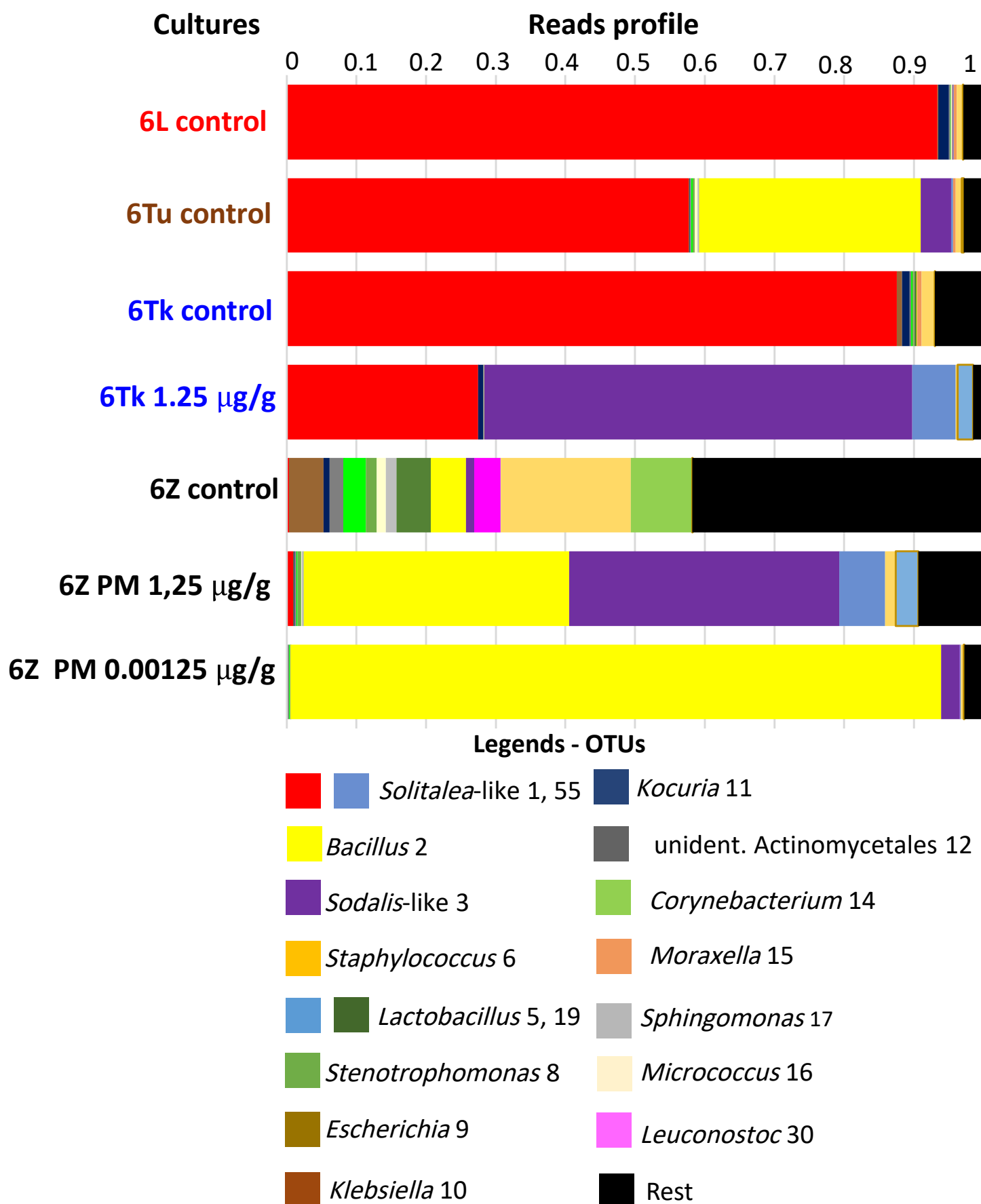

Supplement: Supplementary file 2 — Additional file 2: Fig. S2. Microbiome profiles of the cultures of Acarus siro in the control conditions and under pirimiphos-methyl residues based on barcode sequencing. The columns are means from 6 replicates. [file 12866_2022_2661_MOESM2_ESM.pdf]
